# Genome-wide epigenetic profiling and transcriptome analysis in pediatric Obstructive Sleep Apnea: A focus on Black female children

**DOI:** 10.1016/j.heliyon.2024.e40830

**Published:** 2024-11-29

**Authors:** Bala S.C. Koritala, Sreeja Parameswaran, Omer A. Donmez, Carmy Forney, Hope Rowden, Charles A. Moore, Angela L. Duggins, Alexandra Sestito, Brittany A. Leader, Matthew T. Weirauch, Leah C. Kottyan, David F. Smith

**Affiliations:** a Division of Pediatric Otolaryngology-Head and Neck Surgery, Cincinnati Children's Hospital Medical Center, Cincinnati, OH, USA; b Department of Otolaryngology-Head and Neck Surgery, University of Cincinnati College of Medicine, Cincinnati, OH, USA; c Center for Autoimmune Genomics and Etiology, Cincinnati Children's Hospital Medical Center, Cincinnati, OH, USA; d Warwick Medical School, University of Warwick, Coventry, UK; e Divisions of Biomedical Informatics and Developmental Biology, Cincinnati Children's Hospital Medical Center, Cincinnati, OH, USA; f Division of Human Genetics, Cincinnati Children's Hospital Medical Center, Cincinnati, OH, USA; g Division of Allergy and Immunology, Cincinnati Children's Hospital Medical Center, Cincinnati, OH, USA; h Department of Pediatrics, University of Cincinnati College of Medicine, Cincinnati, OH, USA; i Division of Pulmonary Medicine and the Sleep Center, Cincinnati Children's Hospital Medical Center, Cincinnati, OH, USA

## Abstract

Obstructive Sleep Apnea (OSA) is a common sleep-related breathing disorder characterized by airway obstruction during sleep. Diagnosing pediatric OSA is challenging, particularly in underrepresented populations, leading to disparities in treatment and long-term negative health outcomes. Our study aimed to identify alternative diagnostic tools by investigating genome-wide epigenetic changes and associated transcriptomic alterations in Black female, pediatric patients with OSA. Whole-genome bisulfite sequencing and RNA sequencing were performed on saliva samples from healthy controls and children with OSA. Analysis of differential methylation and gene expression patterns revealed dysregulated inflammation and metabolism pathways in children with OSA. Chromosomes 19 and 22 exhibited elevated methylation signatures in this patient population. Integration of methylation and gene expression data identified specific molecular markers, including *NAP1L4*, *CCR1, and LIF.* The study emphasizes the need to consider both genetic and environmental factors in pediatric OSA, and the identified markers may offer avenues for further research.

## 1 Introduction

Obstructive Sleep Apnea (OSA) is a sleep-related breathing disorder where the airway collapses during sleep, leading to breathing interruptions. Approximately 5 % of children experience OSA, and diagnosis in pediatric patients poses unique challenges [[1], [2], [3]]. If left undiagnosed, OSA may result in behavioral problems and various health complications, such as hypertension, inflammation, endothelial dysfunction, insulin resistance, and obesity [[4], [5], [6]]. Polysomnography (PSG), or overnight sleep study, is the gold standard test used for diagnosis of OSA in both children and adults [7]. However, conducting pediatric sleep studies requires a child-friendly environment, and is typically performed at specialized sleep centers by professionals with extensive experience in diagnosing pediatric sleep disorders. Few pediatric sleep centers are equipped with the necessary resources for accurate sleep studies in children [[8], [9], [10], [11]]. Consequently, diagnosing OSA in children from underrepresented groups is even more challenging. The economic impact of undiagnosed and untreated OSA costs the United States around $150 billion annually [12,13]. Hence, it is essential to develop alternative diagnostic tools that can address the geographical and financial obstacles hindering the diagnosis and treatment of OSA in vulnerable groups.

Pediatric OSA is a complex disease with a spectrum of phenotypic features and is likely influenced by multiple genetic loci and environmental factors [14]. Although the exact etiology of OSA is poorly understood, risk factors including a family history of OSA, obesity, and exposure to second-hand smoke may contribute to the development of OSA in children [15,16]. Racial disparities have also been noted, with Black children being more likely to have severe OSA [[17], [18], [19], [20], [21]]. Previous studies have attempted to identify genetic associations with OSA [[22], [23], [24]], but the interaction between all of these genetic and environmental factors is not yet clearly understood. Understanding these intricate relationships is important for making advances in non-invasive diagnostics and treatment of pediatric OSA. In this particular domain, epigenetics provides important cues as to how epigenetic modifications are involved in the pathogenesis of OSA, considering that its development as well as its molecular pathways represent a logical link between genetics and environment [25]. DNA methylation, a common epigenetic modification, is affected by genetics, environment, and disease state [26]. Methylation is a vital mechanism to control gene expression, influencing which genes are turned on or off and to what degree they produce protein. We hypothesize that comprehensive integrated analysis of methylation/gene expression data in children with OSA could be a potential source of strong OSA marker candidates for diagnosis in pediatric disorder and associated comorbidities.

Contemporary diagnostics are relying more and more on genetic tests for early detection of diseases [27]. Epigenetic principles and changes in gene expression have been found to enhance the diagnosis of several pediatric diseases such as asthma, childhood leukemia, obesity, and metabolic disorders [28]. To our knowledge, our study is the first to examine genome-wide epigenetic and subsequent gene expression changes in the context of OSA in children. Using DNA and RNA isolated from the saliva of Black female, pediatric patients with OSA and race/sex-matched controls, we performed whole-genome bisulfite sequencing and RNA sequencing. By comparing differences in methylation and gene expression profiles, we identified molecular markers that are differentially methylated and expressed in these patients. These omics technologies, from an integrative perspective, could be used for the identification of molecular markers [29] for the diagnosis of OSA. Further investigation is required to fully explore the potential of this approach in predicting this disease.

To facilitate sample collection and ensure high compliance among pediatric patients, we selected saliva as the preferred sample type for DNA and RNA isolation. Saliva was chosen for its non-invasive nature, which is particularly important for pediatric patients, ensuring higher compliance and ease of collection compared to other sample types, including blood draw. Saliva provides a rich source of high-quality DNA and RNA, reflecting both oral and systemic health. This choice supports the study's goals of minimizing discomfort, enhancing participant compliance, and obtaining comprehensive genetic and transcriptomic data.

## 2 Materials and methods

### 2.1 Ethics and study design

Physicians and clinical coordinators from the Division of Pediatric Otolaryngology-Head and Neck Surgery at Cincinnati Children's Hospital Medical Center (CCHMC) oversaw participant recruitment. The study protocol was reviewed and approved by the Institutional Review Board (IRB-2018-3382) at CCHMC. Parental consent was obtained for all enrolled subjects before any procedure or data were recorded. Additionally, informed consent for participation in the study was obtained for each child by explaining the study goals, their medical history, and the study design to at least one parent of each participant.

### 2.2 Subject selection

**Supplementary Fig. 1:**
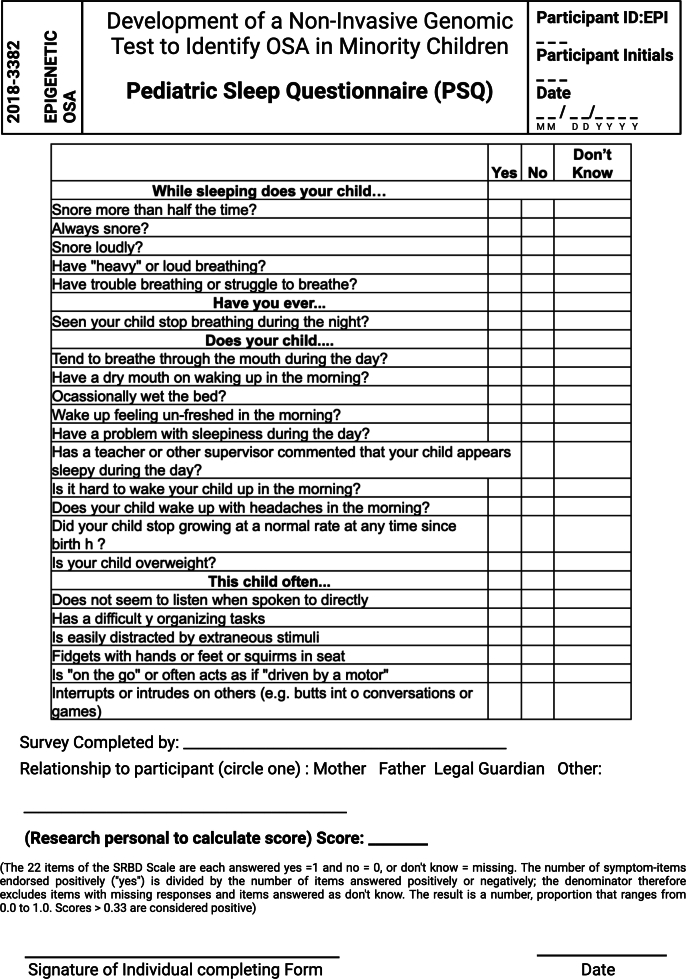
Pediatric Sleep Questionnaire.

**Supplementary Fig. 2:**
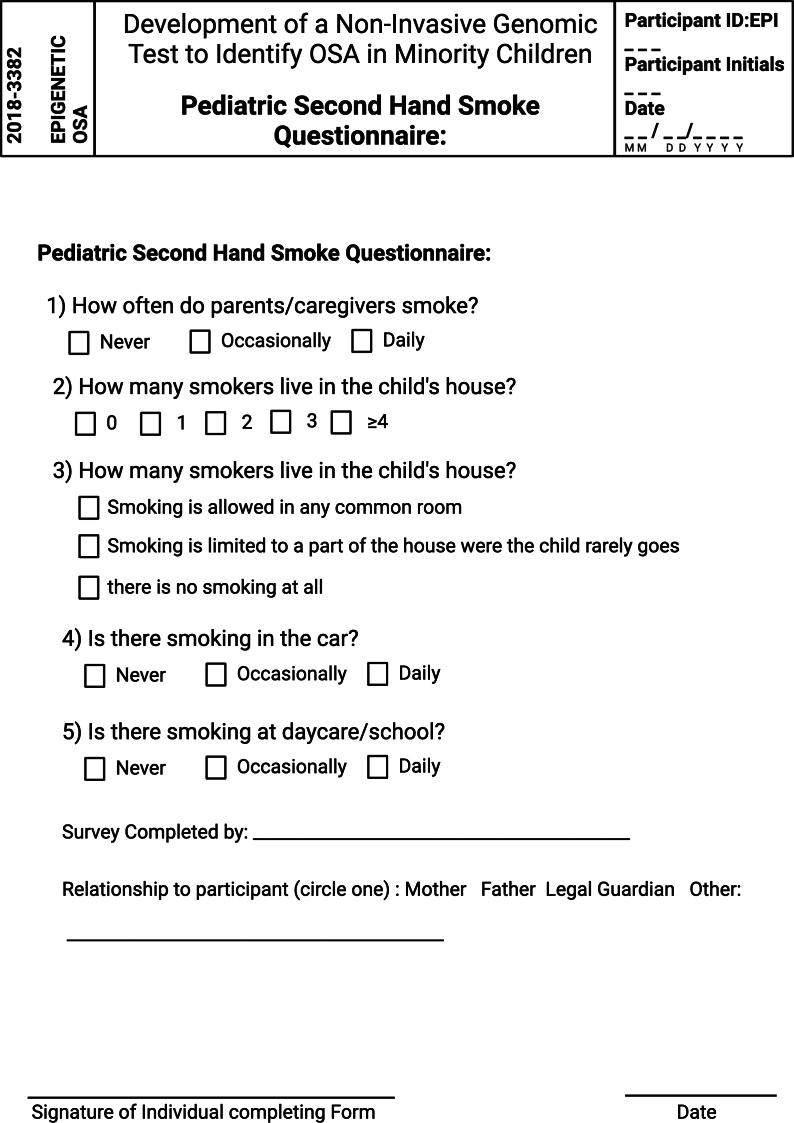
Pediatric Second Hand Smoke Questionnaire.

We enrolled 19 Black female children, including 10 healthy donors, and 9 Obstructive Sleep Apnea (OSA) patients diagnosed by pediatric Polysomnography (PSG) (Table 1). Participants were eligible if they were 3–11 years of age with a Body Mass Index (BMI) Z score of 2.6 or less. Certain conditions led to the exclusion of potential participants, including children who snored in the sleep study without OSA, and those with a history of chronic or recurrent tonsilitis, lung, and heart diseases, neuromuscular or developmental disorders, developmental delay, chronic kidney diseases, endocrine diseases, acute or chronic inflammatory diseases, or any developmental deformity that hindered their capability to make independent decisions. Additionally, a control group using the PSQ (Supplementary Fig. 1) was excluded for any evidence of sleep-related breathing disorder. A questionnaire on exposure to secondhand smoke (Supplementary Fig. 2) was completed by all participants in the study; children with exposure were excluded from the study.

**Table 1:** Polysomnography analysis of Black female children with OSA.

Study ID	BMI (Z-score)	Total Sleep (h)	Sleep efficiency (%)	AHI
EPIA01	0.9	7.6	90.0	4.0
EPIA02	0.6	8.2	94.8	1.0
EPIA03	1.1	6.9	89.9	2.3
EPIA04	0.5	10.1	96.6	3.5
EPIA05	2.6	7.2	91.4	2.5
EPIA06	0.4	7.4	93.3	2.9
EPIA07	2.2	8.6	89.8	1.7
EPIA08	1.3	6.7	74.0	1.9
EPIA09	2.6	6.1	74.6	10.9

### 2.3 Sample collection

Saliva samples were collected from both healthy controls and children with OSA using the Oragene saliva kit (P-182) from DNA Genotek, Inc. Healthy control samples were collected at the clinic, and OSA samples were collected on the day of surgery in the pre-operative suite, just prior to adenotonsillectomy. The specialized buffer solution in the kit was used to stabilize the DNA and RNA from the saliva and prevent bacterial growth. Following collection, the samples were stored at −20 °C until processing for DNA and RNA extraction.

### 2.4 Extraction of nucleic acids

To extract DNA, a 20 μl PrepIT.L2P reagent was added to 0.5 ml of the incubated saliva sample, which had been incubated at 50 °C for an hour. The mixture was then vortexed for 20 s and left on ice for 10 min. After centrifuging the sample at 13,000 ×*g* for 5 min at room temperature, 0.6 ml of 100 % ethanol was added to the supernatant. The mixture was allowed to sit at room temperature for another 10 min to precipitate the DNA. Subsequently, the samples were centrifuged again at 13,000 ×*g* for 2 min at room temperature. The resulting DNA pellets were rinsed with 250 μl of ethanol and dissolved in 50 μl of TE buffer. The extracted DNA was stored at −20 °C for further use.

For RNA purification, 20 μl of neutralizer solution was added to 0.5 ml of the incubated sample at 90 °C for 15 min. The mixture was then incubated on ice for 10 min. After centrifugation at 13,000 ×*g* for 3 min at room temperature, the supernatant was collected and mixed in a 1:1 ratio with RNeasy RLT buffer and ethanol. The mixture was then vortexed six times before RNA purification, following the manufacturer's instructions for the Qiagen RNeasy purification procedure.

The quality and quantity of DNA were assessed using a gDNA kit with the Agilent TapeStation. After clean-up, the quality of DNA was measured using a NanoDrop. RNA quality and quantity were measured on the Agilent Bioanalyzer using the Nano6000 kit. The average RNA integrity value was 8.21, and an average DNA purity of 1.8 was achieved among the samples used for sequencing. These values for each sample are provided in Supplementary Table 1.

### 2.5 Whole genome bisulfite sequencing (WGBS)

Genomic DNA (gDNA) which was extracted was shipped to Beijing Genome Institute (BGI) Hong Kong, China, for bisulfite conversion, library construction, and Illumina sequencing. For this purpose, sonication is used to fragment the DNA, reducing the fragment size to an average size of 100–300 base pairs. After that, end-repair was performed, and methylated sequencing adapters were added to the 3′ end of DNA fragment. We also used the EZ DNA Methylation-Gold kit from Zymo Research (California, USA) for bisulfite conversion of non-methylated DNA. Finally, paired-end libraries were created and sequenced on an Illumina HiSeq 2000 platform. FastQC (version 0.11.7) (http://www.bioinformatics.babraham.ac.uk/projects/fastqc/) was employed in the initial quality control check of the WGBS sequence data, and any bases not of high quality was removed using Trim Galore (version 0.6.0) (https://www.bioinformatics.babraham.ac.uk/projects/trim_galore/). Cutadapt (version 2.1.0) was used to remove the adapters. Subsequently, after these pre-alignment quality control steps, we utilized the Bismark computational tool (v0.18.2) (https://www.bioinformatics.babraham.ac.uk/projects/bismark/) to map bisulfite-converted sequence reads and determine cytosine methylation states. Bismark utilizes Bowtie2 (version 2.3.4.1) for alignment to the human genome (build hg19). The same location reads of the specific strand are than removed using the SAMtools (version 1.9.0) [30] functionality of the Bismark deduplication. MethylKit (version 1.8.1 in R version 3.5.3) was used to identify differentially methylated regions (DMRs) [31]. A threshold of *q* ≤ *0.01* was used to identify DM regions by including a minimum of 25 % methylation difference.

### 2.6 RNA sequencing

RNA sequencing using Illumina sequencing technology was performed by the Beijing Genome Institute (BGI) in Hong Kong. For the preparation of the samples, mRNA was isolated with oligo(dT)-attached magnetic beads. This was followed by fragmentation of the mRNA molecules and reverse transcription with random hexamer priming to generate cDNA, which was then used to synthesize the second-strand cDNA. The synthesized cDNA fragments were repaired and adenylated at the 3′ ends. Then, adapters were ligated to the adenylated cDNA fragments, which were subjected to PCR amplification to amplify the cDNA fragments with adapters. PCR products were purified by AMpure XP Beads with EB solution to be eluted. The Agilent Technologies 2100 bioanalyzer was utilized to evaluate the quality of the library. The library was created by heat denaturing and circularizing the double-stranded PCR products using a splint oligo sequence and then sequencing. The single-strand circular DNA (ssCir DNA) was used as the final library. The library was amplified by the phi29 DNA polymerase, transforming the library into DNA nanoballs (DNBs) - each nanoball has more than 300 copies of a single molecule. The DNBs were captured on a patterned nanoarray and sequenced with paired-end 150-base reads using combinatorial Probe-Anchor Synthesis (cPAS) technology.

We processed the RNA sequencing data using the nf-core/rnaseq pipeline (version 3.8.1) [32]. All raw sequencing data were first quality controlled using FastQC (version 0.11.9) to evaluate global data quality. In the FastQC results, we aimed for %GC content of 45–55 %, base quality scores above 30 per base, and a read distribution balanced with respect to per base sequence content (A & T are approximately equal; G & C are approximately equal). Reads were filtered and trimmed for low-quality bases and adapter sequences using Cutadapt (version 3.4) [33]. The STAR aligner (version 2.7.10a) was used to correctly map the reads to a reference genome after trimming [34]. Putative non-target contaminant ribosomal RNA sequences were then excised from the aligned data with SortMeRNA (version 4.3.4) [35]. Alignments were then sorted and indexed with SAMtools (verson 1.15.1) [30] for retrieval and efficient storage of alignment information. Subsequently, the quality of the RNA sequencing data was further assessed by RseqQC (version 3.0.1) [36], Qualimap (version 2.2.2-dev) [37], dupRadar (version 1.18.0) [38], and Preseq (version 3.1.1). These tools offer thorough evaluations of multiple quality metrics, including read distribution, GC content, duplication rates, and library complexity, to ensure the reliability of data for subsequent analysis. For the differential gene expression analysis, we considered only the samples that passed our rigorous QC steps (samples from 7 patients with OSA and 6 healthy control). All samples passed these rigorous QC steps. For transcript quantification, Salmon (version 1.5.2) [39], a specialized tool for RNA-seq data, was utilized to estimate the transcript expression levels. Salmon employs an accurate and efficient method for quantification, allowing for robust analysis of gene expression patterns. Differential gene expression analysis was performed using DESeq2 (version 1.30.1) [40], a widely used tool for identifying genes with significant expression changes between different conditions or groups. DESeq2 incorporates rigorous statistical models and methods to account for the inherent variability in RNA-seq data. Differential expression was calculated with thresholds of a greater than 2-fold change and an p-value threshold of less than *0.01*.

### 2.7 Enrichment analysis

Gene set enrichment analysis (GSEA) was performed to identify dysregulated hallmark pathways [41,42] and associated regulated targets [[43], [44], [45], [46]] among pediatric patients with OSA. This method offers insights into mechanisms underlying pediatric OSA and potential therapeutic targets. Differential gene expression (DESeq2) data was used as the input for GSEA analysis, and statistical significance was determined by *p* ≤ *0.01*. The Molecular Signatures Database (MSigDB), as a principal component of the hallmark pathways in GSEA, was utilized for this analysis. Furthermore, an enrichment analysis using MSigDB [41] was conducted for genes significantly differentially expressed and methylated within 200,000 bases (distal) of the transcription start site (TSS) using Enrichr [47]. A statistical significance threshold of *p* ≤ *0.05* was applied to identify pathways associated with methylation and gene expression in children with OSA.

### 2.8 Data integration

We used BEDTools intersect [48] to identify the methylation patterns located within either 5000 bases (proximal) or 200,000 bases (distal) of the transcription start site (TSS) of differentially expressed genes.

## 3 Results

**Fig. 1:**
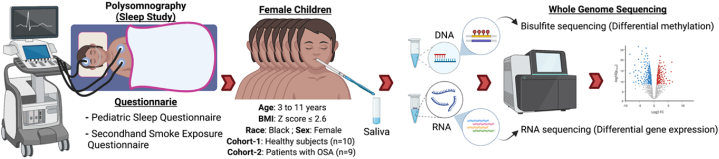
Study design. We recruited a total of 19 female participants, comprising 10 healthy individuals and 9 patients with OSA from the Black population. The age range of the participants was between 3 and 11 years, with a BMI z-score ≤2.6. Participant selection was based on the results of a pediatric sleep questionnaire, a secondhand smoke exposure questionnaire, and/or a sleep study. Polysomnography was employed to measure various sleep parameters, including sleep efficiency, total sleep time, and severity of OSA among the patient group. Salivary samples were collected from all participants, and DNA and RNA were extracted for subsequent whole-genome bi-sulfite and RNA sequencing analysis. This figure was created with BioRender.com.

Children with OSA are unlikely to develop cardiovascular disease at a young age; however, changes in blood pressure [49] and other risk factors can increase their risk for developing hypertension, metabolic syndrome, and cardiovascular diseases in adulthood [50,51]. Obesity and cardiovascular disease are more prevalent among Black populations [52,53], which also have a higher prevalence of severe OSA compared to other ethnic groups [54]. Cultural and financial barriers play a major role in treatment disparities among patients with OSA. In particular, Black populations are less likely to receive diagnosis and treatment of OSA [54]. Therefore, it is important to find alternative and feasible diagnostics to improve OSA diagnosis in this patient population. In the current study, we evaluated both transcriptome and epigenetic changes and associated biological processes among Black female, pediatric patients to develop robust biomarkers for OSA diagnosis (Fig. 1).

### 3.1 Pediatric OSA in black females is associated with disrupted inflammation, metabolism, and DNA repair pathways

**Fig. 2:**
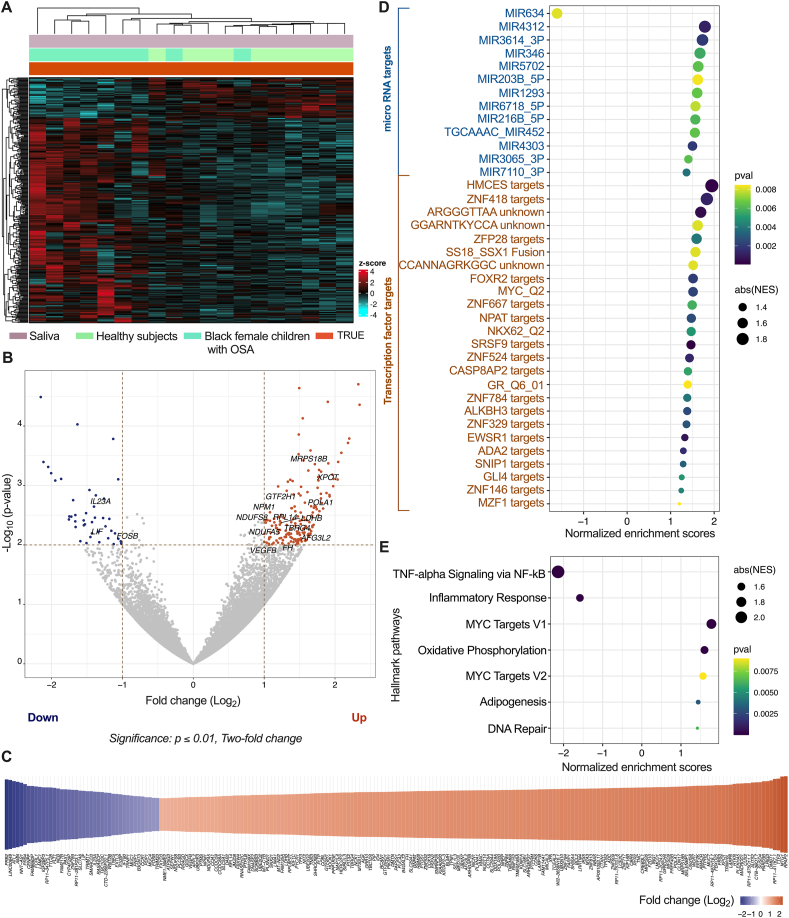
Differential gene expression analysis. **(A)** Clustered heatmap represents transcriptome data collected from saliva samples of both healthy subjects and Black female children with OSA. **(B)** Differential gene expression in children with OSA compared to healthy subjects. The statistical threshold of *p* ≤ *0.01* with a minimum two-fold difference was used to identify significantly up- and down-regulated genes. **(C)** Heatmap of significantly altered genes in children with OSA, and associated fold change compared to healthy subjects. **(D)** Gene Set Enrichment Analysis (GSEA) of regulatory targets associated with differential gene expression. Significantly associated targets with *p* ≤ *0.01* are shown. **(E)** GSEA of hallmark pathways associated with differential gene expression. Enriched GSEA hallmark pathways with *p* ≤ *0.01* are displayed.

To evaluate the biological pathways affected in pediatric patients with OSA, we conducted RNA sequencing analysis on saliva samples collected from both healthy female children and female patients with OSA (Fig. 2A). We employed a statistical significance threshold (*p* ≤ *0.01*) and a minimum two-fold difference to identify differential transcriptome patterns. Our RNA sequencing analysis revealed a total of 12,797 genes expressed above baseline levels in the dataset (Supplementary Table 2). Out of these genes, we observed that 1.66 % (213 genes) showed significant upregulation (171 genes) or downregulation (42 genes) in patients with OSA compared to healthy children (Fig. 2B–C, Supplementary Table 2). To gain a deeper understanding of the underlying mechanisms, we performed an additional analysis using Gene Set Enrichment Analysis (GSEA) [41] to identify the potential regulators driving the observed differential gene expression. Our analysis revealed that multiple microRNA (MIR) and transcription factor targets were significantly (*p* ≤ *0.01*) associated with an upregulation of gene expression, with the exception of *MIR634*, which was associated with downregulation of gene expression (Fig. 2D–Supplementary Table 3). Furthermore, we investigated the GSEA hallmark pathways related to the observed differential transcriptome changes in pediatric patients with OSA. Our analysis indicated a significant (*p* ≤ *0.01*) downregulation of TNF-α signaling via NF-kB and inflammatory responses. Conversely, we observed upregulation in the MYC targets, oxidative phosphorylation, adipogenesis, and DNA repair pathways (Fig. 2E–Supplementary Table 4). These findings shed light on the dysregulation of key pathways in pediatric OSA and provide valuable insights into potential diagnostic or therapeutic targets for mitigating the adverse effects of this condition.

### 3.2 Elevated methylation signatures in chromosome 19 and 22 among black females

**Fig. 3:**
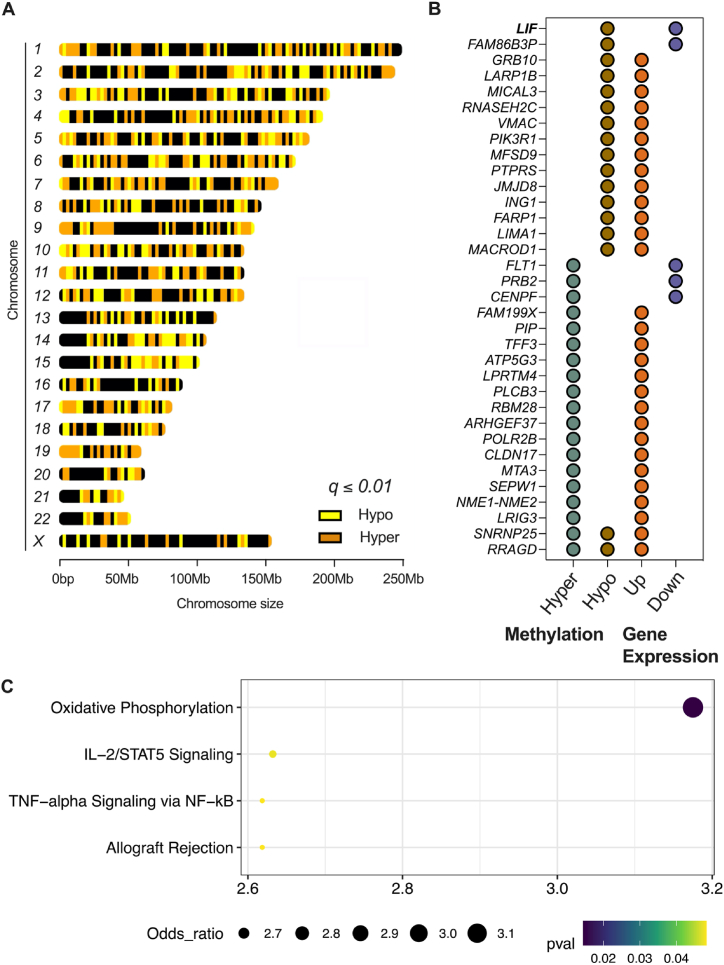
Differential methylation analysis. **(A)** Differentially hyper- and hypo-methylated regions across the chromosomes of Black female children with OSA, compared to matched healthy subjects. To identify differential methylated regions, a threshold of *q* ≤ *0.01* and a minimum methylation difference of 25 % were employed. **(B)** Genes that exhibit differential gene expression (*p* ≤ *0.01* with a minimum two-fold difference) and differential methylation (*q* ≤ *0.01* with a minimum of 25 % difference) within a 5000 bases (proximal) of their TSS. **(C)** MSigDB enrichment analysis of genes significantly differentially expressed and methylated within 200,000 bases (distal) of their TSS.

DNA methylation is a crucial regulatory mechanism in disease processes, offering promise for therapeutic intervention due to its reversible nature and potential heritability [26]. Notably, the diagnostic complexities associated with pediatric OSA [[1], [2], [3]] underscore the importance of assessing DNA methylation. However, limited research has been conducted on epigenetic signatures in pediatric OSA, with available studies focused on specific targets [55,56]. To bridge this gap, we aimed to comprehensively analyze whole-genome methylation patterns in Black female, pediatric patients with OSA using whole-genome bi-sulfite sequencing. We employed a *q* ≤ *0.01* threshold with percent methylation differences larger than 25 % and identified 40,193 hyper- and 43,631 hypo-methylated regions across all 22 pairs of autosomes and the X chromosome (Fig. 3A, Table 2). No methylation patterns were observed on the Y chromosome due to our focus on females. Interestingly, chromosome 19 exhibited a higher percentage of hyper-methylated regions (0.0000422804 %) compared to other chromosomes, while chromosome 22 had a higher percentage of hypo-methylated regions (0.0000370337 %). These findings contribute to a better understanding of the epigenetic landscape in pediatric OSA and provide a foundation for further research.

**Table 2:** Methylation percentage in each chromosome of Black female children with OSA.

Chromosome	Hyper methylated (%)	Hypo methylated (%)
1	1.68E-05	1.56E-05
2	1.76E-05	2.05E-05
3	1.41E-05	2.32E-05
4	2.19E-05	1.62E-05
5	2.10E-05	2.81E-05
6	2.33E-05	2.10E-05
7	3.45E-05	1.57E-05
8	1.36E-05	1.70E-05
9	1.69E-05	2.76E-05
10	1.84E-05	1.99E-05
11	1.85E-05	2.07E-05
12	1.71E-05	1.56E-05
13	8.68E-06	1.56E-05
14	1.49E-05	2.23E-05
15	1.07E-05	2.92E-05
16	8.85E-06	1.32E-05
17	2.95E-05	3.32E-05
18	1.53E-05	1.66E-05
19	4.22E-05	1.18E-05
20	1.42E-05	2.22E-05
21	2.07E-05	1.86E-05
22	1.36E-05	3.70E-05
X	7.08E-06	9.01E-06

### 3.3 Identification of potential biomarkers in black female, pediatric patients with OSA

The integration of various biological datasets, such as epigenetic and gene expression information, holds tremendous potential for advancing our understanding of the molecular characteristics associated with pediatric OSA in distinct ethnic populations. We focus here on the examination of DNA methylation patterns located within either 5000 bases (proximal) or 200,000 bases (distal) of the TSS of differentially expressed genes. These distances correspond to DNA alterations to promoters or enhancers, respectively. This approach facilitated the identification of specific putative gene targets with significant alterations in both methylation and expression levels. Within a 5 kilobase pair range from the TSS, we discovered 34 gene targets with significant changes in both methylation and gene expression. Among these targets, 85.3 % of the genes showed upregulation, while only 14.7 % showed downregulation. Seven genes (*SNRNP25, NME1-NME2, SEPW1, MTA3, ATP5G3, ARHGEF37, RRAGD*) had two proximal hyper-methylated sites, and one gene (*MFSD9*) had four hypo-methylated sites (Fig. 3B**–**Supplementary Table 5). Within the more lenient range of 200 kilobase pairs from the TSS, we identified 197 gene targets that demonstrated significant changes in both methylation and gene expression. Among these targets, 80.2 % were associated with upregulation, while only 19.8 % were downregulated. *NAP1L4* exhibited a greater number of hyper-methylation sites with upregulation, whereas *CCR1* exhibited a greater number of hypo-methylation sites with downregulation (Supplementary Table 6). We further performed pathway analysis of these 197 gene targets to understand their linkage with pathways potentially involved in OSA. We identified genes associated with the oxidative phosphorylation (*PDP1*, *AFG3L2*, *LDHB, FH, NDUFS8, NDUFA5,* and *NCK1*), IL-2/STAT5 signaling (*MUC1, AMACR, RRAGD, LIF, and BCL2*), TNF-α signaling via NF-kB, (*RCAN1, IL23A, LIF, TNC,* and *FOSB*), and allograft rejection (*CCR1, TPD52, NPM1, LIF, and NCK1*) (Fig. 3C). The recurrent identification of *LIF* in these pathways underscores its potential as a critical gene in the epigenetic regulation of inflammatory and immune responses associated with pediatric OSA. DNA methylation patterns within specified distances from the TSS revealed significant alterations in both methylation and expression levels of many genes. These associated pathways offer valuable insights into the molecular characteristics of pediatric OSA in this ethnic group.

## 4 Discussion

Our study aimed to identify molecular targets useful in diagnosing OSA and understanding the mechanisms driving disease susceptibility in Black female children through analyses of differential methylation and gene expression patterns. Although male predisposition to OSA in children has been reported [57], there is limited evidence regarding gender-specific causative factors, presentation, and treatment outcomes of OSA. In females, symptom manifestation might vary; for example, they may experience restlessness or mood changes, in contrast to symptoms usually seen in males, such as snoring and daytime sleepiness [58]. Furthermore, hormonal changes in females during puberty can affect OSA severity and presentation, making it important to refine diagnostic criteria and treatment approaches. We assessed DNA and RNA from saliva samples of Black female healthy children and patients with OSA using whole-genome bisulfite and RNA sequencing. We identified dysregulated inflammation, metabolism, and DNA repair pathways in pediatric OSA, as well as elevated methylation signatures in chromosomes 19 and 22 in our cohort. We have identified molecular markers of pediatric OSA by integrating methylation and gene expression data. To our knowledge, this is the first study that has broadly investigated genome-wide changes in epigenetics and their associated transcriptomic alterations in pediatric OSA.

OSA is a complex disease influenced by both genetic and environmental factors [59]. Traditional diagnostic methods, such as PSG, are resource-intensive and not easily accessible, particularly for underrepresented groups. Therefore, the development and application of alternative diagnostic tools are essential. Utilizing an epigenomic approach combined with gene profiling creates a potential method to identify effective biomarkers for OSA diagnosis and treatment responses in clinical practice.

RNA sequencing analysis revealed differential gene expression patterns in those with OSA compared to healthy children. Dysregulation of TNF-α signaling via NF-κB and inflammatory responses suggests an underlying inflammatory component in pediatric OSA. This finding aligns with previous research linking OSA to chronic inflammation and the development of various health conditions, including cardiovascular diseases and metabolic disorders [60,61]. Upregulation of MYC Targets, Oxidative Phosphorylation, Adipogenesis, and DNA repair pathways also represents the molecular functions affected in pediatric OSA, providing potential drug target candidates capable of attenuating developmental impacts, and improving treatment efficacy.

Whole-genome bisulfite sequencing analysis is identified increased methylation signatures on chromosomes 19 and 22 among Black female children with OSA. The differential methylation patterns observed in these chromosomes provide valuable insights into the epigenetic landscape in this specific population of children with OSA. These findings are especially important given that this study was conducted in Black pediatric patients, a group with a higher prevalence of severe OSA and notable racial disparities in OSA diagnosis and treatment. Understanding the epigenetic mechanisms involved in these processes can help enable more targeted diagnoses.

Our study combined methylation and gene expression data to identify the key marker molecules and pathways of pediatric OSA. The integration of DNA methylation changes within a fixed region around the TSS enabled the identification of gene targets that showed significant changes in methylation and gene expression levels. Specifically, these gene targets are involved in metabolic and inflammatory pathways, including oxidative phosphorylation, IL-2/STAT5 signaling TNF-α signaling via NF-kB, and allograft rejection. Follow-on studies and further validation would be required to assess the diagnostic and prognostic potential of these markers.

In conclusion, this study enhances the characterization of pediatric OSA molecular features, leading to a better understanding of OSA in a patient population that faces substantial challenges in terms of healthcare system compliance for diagnosis and the availability of treatment providers. The results point to the necessity of considering both genetic and environmental factors and emphasize the need for alternative and affordable diagnostic solutions for underprivileged populations in this disease process.

## 5 Limitations of the study

The current study offers valuable insights into pediatric OSA within a specific cohort, providing a foundation for future research and advancing our understanding of this condition. Given that our intent is to generate tools that could be used to develop diagnostics for an important underrepresented population, our study was limited to Black female pediatric participants. However, our findings should be considered within the context of our relatively small sample size. Larger, more diverse cohorts are needed in future studies to demonstrate generalizability to other populations, sexes, and age groups. Expanding the age range beyond 3–11 years will enable us to capture the full complexity and variability of pediatric OSA in both older and younger children. One notable aspect of our study is the non-invasive approach we employed in developing diagnostics, utilizing saliva samples from the pediatric population. This novel method prepares the ground for subsequent functional probing of selected potential molecular markers and its clinical utility in defining the molecular signature of OSA aimed at assisting in the diagnostic and therapeutic personalized approaches to OSA. Although the cross-sectional design of our study limits our ability to establish causality or determine the temporal sequence of events, future longitudinal studies are needed to address these issues. While the present study sheds light on previously unknown information, its limitations, and the need for replication work in other ethnic populations, such efforts could provide improved findings in the field and potentially contribute to the knowledge expansion of pediatric OSA.

## CRediT authorship contribution statement

**Bala S.C. Koritala:** Data curation, Formal analysis, Investigation, Methodology, Supervision, Validation, Writing – original draft, Writing – review & editing. **Sreeja Parameswaran:** Data curation, Formal analysis, Methodology, Writing – review & editing. **Omer A. Donmez:** Formal analysis, Investigation, Methodology, Writing – review & editing. **Carmy Forney:** Investigation, Methodology, Writing – review & editing. **Hope Rowden:** Investigation, Writing – review & editing. **Charles A. Moore:** Conceptualization, Investigation, Writing – review & editing. **Angela L. Duggins:** Investigation, Writing – review & editing. **Alexandra Sestito:** Investigation, Writing – review & editing. **Brittany A. Leader:** Conceptualization, Writing – review & editing. **Matthew T. Weirauch:** Funding acquisition, Supervision, Writing – review & editing. **Leah C. Kottyan:** Conceptualization, Funding acquisition, Supervision, Writing – review & editing. **David F. Smith:** Conceptualization, Funding acquisition, Investigation, Supervision, Writing – review & editing.

## Data availability statement

The associated raw and analyzed data files are available at NCBI's Gene Expression Omnibus (GEO accession number: GSE237282).

## Declaration of competing interest

The authors declare that they have no known competing financial interests or personal relationships that could have appeared to influence the work reported in this paper.

## Appendix A Supplementary data

Supplementary Table 1 Quality of DNA and RNA. Supplementary Table 1

Supplementary Table 2 DESeq2 analysis of RNA sequencing Supplementary Table 2

Supplementary Table 3 GSEA analysis of regulatory targets Supplementary Table 3

Supplementary Table 4 GSEA analysis of hallmark pathways Supplementary Table 4

Supplementary Table 5 Genes with differential gene expression and methylation from 5000 bases (proximal) of their TSS Supplementary Table 5

Supplementary Table 6 Genes with differential gene expression and methylation from 200,000 bases (distal) of their TSS Supplementary Table 6
